# The Importance of Inhaler Adherence to Prevent COPD Exacerbations

**DOI:** 10.3390/medsci7040054

**Published:** 2019-04-01

**Authors:** Jose R. Jardim, Oliver A. Nascimento

**Affiliations:** 1 Respiratory Division, Escola Paulista de Medicina/Universidade Federal de São Paulo (EPM/Unifesp), São Paulo 04023-062, Brazil; olivernascimento@yahoo.com.br; 2 Pulmonary Rehabilitation Unit, EPM/Unifesp, São Paulo 04023-062, Brazil; 3 Regional Medical Expert at GlaxoSmithKline (GSK), Rio de Janeiro 22783-110, Brazil; 4 Faculdade de Medicina São Leopoldo Mandic/Instituto São Leopoldo Mandic, Campinas 13045-755, Brazil; 5 Internal Medical Expert at GlaxoSmithKline (GSK), Rio de Janeiro 22783-110, Brazil

**Keywords:** COPD, inhalers, bronchodilators, inhaled steroid, adherence

## Abstract

It has been shown that the better outcomes of chronic obstructive pulmonary disease (COPD) are closely associated with adherence to drug therapy, independent of the treatment administered. The clinical trial Towards a Revolution in COPD Health (TORCH) study clearly showed in a three year follow up that patients with good adherence to their inhaler treatment presented a longer time before the first exacerbation, a lower susceptibility to exacerbation and lower all-cause mortality. The Latin American Study of 24-h Symptoms in Chronic Obstructive Pulmonary Disease (LASSYC), a real-life study, evaluated the self-reported inhaler adherence in COPD patients in seven countries in a cross-sectional non-interventional study and found that approximately 50% of the patients had good adherence, 30% moderate adherence and 20% poor adherence. Adherence to inhaler may be evaluated by the specific inhaler adherence questionnaire, the Test of Adherence to Inhalers (TAI). Several factors may predict the incorrect use of inhalers or adherence in COPD outpatient, including the number of devices and the daily dosing frequency. Ideally, patient education, simplicity of the device operation, the use of just one device for multiple medications and the best adaptation of the patient to the inhaler should guide the physician in prescribing the device.

## 1. Introduction

Chronic obstructive pulmonary disease (COPD) is characterized by chronic respiratory symptoms such as cough, phlegm production and dyspnea that have a great impact on the patient due to physical limitation and reduction of the quality of life [1]. In addition, patients with COPD may experience episodes of exacerbation, characterized by worsening of symptoms in relation to their daily variation, which result in additional treatment and may result in hospitalizations and disease progression. Therefore, the exacerbation must be treated as soon as itis diagnosed to reduce the negative impact. The COPD exacerbation treatment is based on systemic corticosteroids, antibiotics (if necessary) and inhaled short action bronchodilators (beta-2 agonist and/or muscarinic antagonist). The Global Initiative for Chronic Obstructive Lung Disease (GOLD) document recommends that maintenance therapy with inhaled long-acting bronchodilators should be prescribed as early as possible, even during the hospitalization and before the discharge. So, the inhaled therapy is very important to treat the acute exacerbation and to prevent the next one [1].

Medication delivery to the lungs is always preferable over systemic administration due to several advantages such as faster action onset, high therapeutic effect and lower systemic adverse events [2,3]. However, there are several factors that influence the optimal treatment of the patient such as patient characteristics (age, conscience, breathing pattern, airway diameter, disease severity, inspiratory flow), aerosol characteristics (inhaler device and inhaled drug characteristics), pharmacokinetics and pharmacodynamics [4]. Patients must be instructed about the device use and how to inhale the medicines properly. However, not all patients will perform in the correct way due to either cognitive or physical reasons, or both. When an inhaler device is not used properly, it affects lung deposition, and thus results in lower efficacy and possibly increased chances of side effects. To improve treatment efficacy and efficiency, it is very important to individualize the inhaler device to specific patient populations and choose the most friendly inhaler for each patient. This review aimed to provide an overview of the current COPD adherence landscape and highlight the importance of medication adherence and the negative impact of non-adherence in COPD patients.

## 2. Definition and Measurement of Adherence

### 2.1. Definition of Adherence

It is not an easy task to define adherence to treatment. In the simplest way, adherence should be defined as how patients follow the medical prescription. However, numerous variables influence medication use, such as the route of administration, frequency of use, taste, response to therapy and adverse events. For example, a single oral medication could be easier to adhere to than treatment with three pills used at different frequency. In addition, inhaled medications have one more variable: the correct use of the device. In addition, there are other variables related to patient’s behaviors and beliefs, and the patient-physician relationship that directly influence adherence. The World Health Organization (WHO) document adopted the definition of adherence to long-term therapy as “the extent to which a person’s behaviour—taking medication, following a diet, and/or executing lifestyle changes, corresponds with agreed recommendations from a health care provider” [5]. The European Society for Patient Adherence, Compliance and Persistence (ESPACOMP) wrote a report that was undertaken within the ABC (Ascertaining Barriers to Compliance) project and it defined adherence to medications as “the process by which patients take their medications as prescribed” [6]. According to the authors, adherence has three components: initiation, implementation and persistence [6,7]. Treatment initiation is when the patient takes the first dose of the medication prescribed by a health care provider (HCP). The therapy implementation is “the extent to which a patient’s actual dosing corresponds to the prescribed dosing regimen, from initiation until the last dose” [7]. This is a longitudinal description of patient behavior over time, that is, his or her dosing history, and this includes the inhaler technique. Persistence “is the length of time between initiation and the last dose, which immediately precedes discontinuation” of the prescribed medication [7]. Therefore, if the patient delays the medication initiation, does not do the correct inhaler technique and/or interrupts his/her treatment early, there is inadequate adherence, non-adherence or poor adherence to therapy. The World Health Organization (WHO) described three forms of non-adherence: erratic non-adherence, unwitting non-adherence and intelligent non-adherence [5]. Erratic non-adherence is unintentional and related to a patient’s inability to adjust treatment to their daily routine (troubled routine or busy schedules) and/or associated to forgetfulness [3]. Unwitting non-adherence is also an unintentional non-adherence due to lack of understanding about the therapy and/or importance of the adherence [5]. Intelligent non-adherence is when the patient, intentionally, alters the treatment course, i.e., does not initiate the treatment or discontinues it. Patients who feel better may decide that they no longer need to take prescribed medications. Fear of perceived short- or long-term side-effects of inhaled corticosteroids (ICS) may cause some patients to reduce or discontinue dosing. Patients may abandon the therapy due to fear of side effects, the improvement of their symptoms, bad tasting medication and complex prescriptions. Poor adherence to inhaled drug therapy in individuals with COPD may be associated with suboptimal therapeutic outcomes.

### 2.2. Methods to Measure Adherence

Numerous studies have suggested that patients adhere to inhaled drug therapy less than to orally administered therapies [8,9]. Poor adherence to asthma treatment may be associated with suboptimal outcomes and disease exacerbation [10], and evidence shows that adherence to inhaled therapy for asthma and COPD has the potential to reduce the risk of exacerbations, through increased symptom control, improving outcomes for patients, and reducing healthcare resource utilization [9,10,11]. 

In this point of view, the poorer outcome could be used to evaluate or to diagnose the non-adherence. However, the poor outcome may be due to the disease characteristics rather than the patient adherence. Thus, it is very important to have methods to measure adherence.

There are a lot of methods to monitor adherence that include subjective methods (self-reporting and clinician judgement) and objectives methods (pharmacy register, checking the device number of dose used/canister weight, biochemical measurement, and the use of electronic monitoring devices) [12,13,14].

Subjective methods may be evaluated by patient self-reporting (such as medication diaries or retrospective questionnaires) or by the HCP evaluation. The patient self-report methods to measure adherence are simple and low cost but they depend on patient’s memory (inaccurate) and they significantly over-estimate adherence. Within the patient self-report methods, questionnaires are better, once they are standardized, validated and can provide detailed information about patterns of medication use, patient’s perceptions and barriers to medication use [13,15]. The most common and specific questionnaire that evaluates inhaler non-adherence is the Test of Adherence to Inhalers (TAI). This test includes 12 items and the first five items were designed to identify erratic non-adherence behavior (forgetfulness to take medication) and items from 6 to 10 identify deliberate or intentional non-adherence behavior (the patient decides not to take medications). These first 10 items are self-administered and scored from 1 to 5 (where 1 = worst possible score and 5 = best possible score). The final two items are completed by the HCP and scored as 1 or 2 (where 1 = bad and 2 = good) and they were designed to identify unwitting non-adherent behavior (failure in understanding medication use, dosage or inhalation technique). The total score ranges between 12 and 54 for the 12-item TAI and the higher score is the best adherence [15]. The HCP judgement can be integrated into any clinical interaction, is also simple and low cost and it is capable of assessing adherence and competence. However, it is more time consuming to make a detailed competence assessment and it is equally inaccurate and tends to over-estimate adherence [12].

The objective methods for monitoring adherence are more precise. The direct biochemical measurements, which may be performed in blood, urine or hair are precise and objective. However, they are invasive, not available for all medications, they reflect only a snapshot in time and do not provide information about pattern of use over time, and they are costly [12,13,14]. The evaluation of the number of doses used (dry powder inhaler (DPI) medications) or canister weight (pressured metered-dose inhaler (pMDI) medications) are simple and objective. On the other hand, they are not able to assess competence, device error or detect medication sharing. For the canister weight, equipment and some skill are required [12]. The pharmacy registers and prescription records are simple, objective and cheap. It is possible to evaluate the amount of medication dispensed and returned at the follow up. However, these are dependent on the health service infrastructure and there is limited data about daily patterns of medication use by the patients [12,13]. 

Electronic monitoring (smart inhalers) record the date and time of medication use events, allowing long term monitoring with detailed information about the daily patterns of medication use. They are the gold standard monitoring modality, are able to distinguish adherence from competence and may provide feedback. Although they are objective and precise, they do not confirm the correct use of the device and they are still costly (may change in the future) and depend on technology and infrastructure [12,13].

All methods described above evaluate one part of adherence, however, it is also very important to evaluate device technique used by the patient at each visit.

## 3. Factors Associated with Non-Adherence

Adherence is a preferred term in relation to compliance as the latter infers a lack of patient involvement. Actually, treatment should be an agreement between the physician and the patient involving a concordance of the patient in following the recommendations [16]. Poor adherence is a great concern in COPD patients. Overall, WHO considers that only 50% of patients in chronic therapy are adherent to treatment. Estimates for COPD are not too different from this figure, but it may be higher when patients are followed up by the same healthcare team and treatment education is constantly repeated. Despite the great evolution in COPD treatment, poor adherence remains a major problem for these patients [17], and negatively impacts exacerbation rate, quality of life, and healthcare resources [18]. The proportion of COPD patients with good adherence to prescribed treatment differs greatly between clinical trials (70–90%) and clinical settings (10–40%) [19]. The reasons for the poor adherence in the clinical settings are multifactorial and may be related to [19,20]:

- Patient characteristics (patients’ beliefs, psychological condition, cognitive status, self-efficacy and co-morbidities);
- Social factors (patient-prescriber relationship, access to medication, social support and device training and follow-up);
- Pharmacological treatment (method of administration, inhaler device, dosing regimen, polypharmacy, and side effects).

Despite the classification described above, these factors intersect themselves very frequently, mainly patients and social factors. COPD affects people over 40 years old, however the disease is underdiagnosed in younger patients, with less symptoms and better lung function [21]. The disease progression comes with ageing and the treatment may start in the elderly, when the patients have worse cognitive status and higher prevalence of anxiety and depression. In addition, patients may present other co-morbidities and polypharmacy is very frequent. In a prospective study that evaluated adherence to salmeterol/fluticasone after the patient’s discharge due to an acute COPD exacerbation, it was demonstrated that only 6% of the patients had an actual adherence greater than 80%. The adherence was evaluated by an electronic device that could also study the inhaler technique errors. The major determinants of poor adherence were the presence of cognitive impairment, which affected the patient’s ability to remember to take the medication, and severe hyperinflation, which affected the ability of the individual to generate sufficient inhalation flow and as a consequence resulted in impaired drug delivery [22]. Another study showed that the improvement obtained with the treatment is one factor that leads to non-adherence; about 31% of COPD patients consciously decided not to use their medication if they were better [23].

In general, the pharmacological treatment factors that may increase non-adherence are related to more complex inhaler devices that lead to incorrect inhaler use, higher frequency of daily doses, polypharmacy and side effects. Several factors may predict the incorrect use of inhalers in ambulatory COPD patients, including the number of devices. It has been shown that the mean numbers of errors using just one device is lower than when using three inhalers [24]. A matched cohort study with more than 16,000 COPD patients compared the COPD exacerbations rate incidence and reliever medication use between two cohorts that were formed by one “similar-device cohort” and another “mixed-device cohort”. It was shown that similar-device patients had an 18% reduction in COPD exacerbation rate incidence compared to mixed-device patients and 46% less use of reliever medication [25]. So, the inhaler simplicity may also lead patients to increase adherence and improve outcomes. The daily dosing frequency may also be a barrier to high adherence. A large retrospective study with over 50,000 COPD patients followed up by the proportion of days covered for one year after the initial COPD diagnosis, showed an inverse correlation between the number of inhaled daily doses and adherence: for once a day (QD), twice a day (BID), three times a day (TID) and four times a day (QID), the proportion of days covered were 43.3, 37.0%, 30.2% and 23%, respectively [26]. In addition, there are studies reinforcing the reduction of COPD exacerbations when comparing QD versus BID medications. The QD fluticasone furoate/vilanterol were more effective in reducing exacerbations when compared to other BID inhaled corticosteroid/long acting beta-2 agonist associations in the Salford Lung Study (8.2% additional reduction in exacerbation rate) and once-daily triple therapy (fluticasone furoate/umeclidinium/vilanterol) in the Lung Function and Quality of Life Assessment in COPD with Closed Triple Therapy (FULFIL) trial (35% reduction in exacerbation rate versus BID budesonide/formoterol) [27,28]. In the Effect of Indacaterol Glycopyronium vs Fluticasone Salmeterol on COPD Exacerbations (FLAME) study, the indacaterol/glycopyrronium association reduced the exacerbation rate by 16% when compared to salmeterol/fluticasone [29]. Also, QD triple therapy combination of fluticasone furoate/umeclidinium/vilanterol had more efficacy and reduced the exacerbation rate by 15% when compared to fluticasone furoate/vilanterol and 25% when compared to fluticasone umeclidinium/vilanterol in a sample of high-risk COPD patients [28].

## 4. The Impact of Non-Adherence on Exacerbations, Hospitalizations and Resource Use

It has been shown that better outcomes with treatment are closely associated to adherence to drug therapy, independent of the treatment administered [30]. Usually it is assumed that COPD patients may present better adherence to treatment as they perceive an immediate symptomatic relief [31]. It is also believed that more severe patients tend to be more adherent to treatment [32]. A recent systematic review that evaluated the clinical and economic impact of non-adherence in COPD showed a clear association between non-adherence and increase in hospitalizations and mortality, worse in quality of life and loss of productivity [33]. In addition, the authors related the negative clinical and economic consequences to non-adherent asthmatic and COPD patients [34].

The Study Towards a Revolution in COPD Health (TORCH) evaluated over six thousand patients for three years and investigated the mortality in groups receiving four different regimens of treatment delivered by inhalers. This also allowed the opportunity to study the effect of adherence on severe exacerbations needing hospitalization [35,36]. Overall, approximately 80% of the patients were adherent to all four regimens and in the same proportion in the moderate, severe and very severe stages of the disease. Patients with poorer adherence at baseline were more dyspneic and presented higher airflow limitation. The group with good adherence to treatment had a 44% lower rate of severe exacerbations (around 0.15 to 0.25 per patient per year, respectively) with a rate ratio of 0.56 (95% confidence interval (CI) 0.48–0.65, *p* < 0.001), independent of study treatment and after adjustment for region, sex, age, smoking status, body mass index, prior exacerbation and airflow limitation. In the same way, patients with good adherence showed a longer time for the first exacerbation, demonstrating a lower susceptibility to exacerbation. These results clearly show how adherence to treatment is important to achieve a better outcome. In addition, better adherence was also reflected in the mortality rate: the good adherence group had a lower all-cause mortality rate with a relative risk reduction of 21.2% compared to 6.6% in the poorer adherent group, comparing the groups with salmeterol-propionate fluticasone versus placebo [36]. It is possible that the high adherence rate seen in TORCH is because patients in clinical trials are usually closely supervised and tend to have a higher adherence than in the real life. This may be true, as patients in TORCH allocated to placebo also had a high adherence rate, which raised the term “the healthy adherer effect” that may reflect a surrogate marker to overall healthy behavior, according to the authors [36]. A recent study showed that the group of patients with irregular medication use and with frequent critical technique errors had an increase in mortality compared to patients with regular medication use and without critical technique error (adjusted hazard ratio 8.69 (95% CI 1.82–40.83; *p* = 0.007)) [37].

The LASSYC study in Latin America evaluated the self-reported inhaler adherence in COPD patients in seven countries in a cross-sectional non-interventional study according to the Morisky Medication Adherence Scale (MMAS-8) and the TAI [38]. As in most COPD studies, most were men (60%), aged approximately 70 years old and with moderate to severe disease (forced expiratory volume in one second (FEV1) 50%). According to both questionnaires, approximately 50% of the patients had good adherence, 30% had moderate adherence and 20% had poor adherence. The patients with poorer adherence were associated with lower smoking history and schooling and a higher COPD Assessment Test score (CAT) exacerbation rate in the previous year. This is a lower value than the one found in the TORCH study, however, in a clinical trial such TORCH, patients are regularly and continuously educated and monitored about following the instructions. However, despite the difference in adherence rates in both studies, the patients with lower adherence presented higher exacerbation rates [38].

The higher exacerbation rate is associated with lower adherence and may be a factor that predisposes these patients to higher morbidity, hospitalizations and use of health resources. A cross sectional study of Medicare in the United States retrospectively evaluated in over 30 thousand patients with COPD from a large database, the association between adherence to treatment and hospitalization and use of resources. Medications included steroids associated or not to long acting beta-adrenergic bronchodilators, anticholinergics and xanthine. The database was examined for two years and after adjustments for all covariates, adherence to treatment was significantly associated to lower hospitalization (relative risk (RR) = 0.88, CI 95% 0.85–0.92) and in the same way, patients with higher adherence significantly exhibited less care spending. The authors postulated that two mechanisms could explain these outcomes, one is that adherence to COPD treatment may alleviate symptoms and prevent exacerbation; these patients would also be adherent to their other medications for chronic conditions preventing their acute outcomes. The authors named this condition “healthy behavior” [39]. 

A cross-sectional study evaluated the impact of medication adherence on absenteeism and short-term disability in employees with diabetes, hypertension, dyslipidemia and asthma/COPD. They estimated that adherent patients had between 1.7 and 7.1 fewer days absent from work and between 1.1 and 5 fewer days on short-term disability [40].

## 5. The Importance of Inhaler Devices and Good Inhaler Technique 

After the Montreal Protocol on Substances that Deplete the Ozone Layer in 1987 [41] several different inhaler devices have been developed for treatment of COPD patients such as DPIs (Ellipta^®^, Diskus^®^, Turbuhaler^®^, Aerolizer^®^, Handihaler^®^, Breezehaler^®^, NEXThaler^®^, Pulvinal^®^), a pMDI using a less toxic gas such as hydrofluoroalkane (HFA), a soft mist inhaler (Respimat^®^) with water and nebulizers. Every different inhaler has been developed in the belief that it would fit better a patient needs and would be easier for the patients to use it. All the inhaler devices have advantages and disadvantages (Table 1) and their performance is dictated by the relationship between the drug formulation, device and patient. Understanding these factors and how they influence the treatment are essential for symptom relief and disease control.

The DPI may be more convenient for some patients once they are breath-actuated and do not require coordination to dose release and to inhale as the pMDI does. However, in the devices where a capsule must be inserted for actuation, such as the Breezehaler^®^ and Aerolizer^®^, patients with hand arthrosis may have difficultly using it appropriately and a more appropriate device should be recommended for them. The same may occur with the soft mist inhaler.

Errors during the inhaler use that affect the dose delivery to the lungs are called critical errors and some devices present less critical errors. Usually the number of errors considered of greatest importance to the effectiveness of treatment a patient is greater for MDIs than for DPIs [42]. In a recent study, COPD and asthma patients were evaluated about the correct inhale technique after reading the patient information leaflet. The results showed that fewer COPD patients made critical errors using Ellipta^®^ compared to other inhalers (Turbuhaler^®^, pMDI, Breezehaler^®^, Diskus^®^ and Handihaler^®^). In addition, more asthma and COPD patients preferred Ellipta to other inhalers [43]. In addition, in another study head to head comparing the efficacy of two long-acting muscarinic antogonists/long-acting beta2 agonists LAMA/LABA combinations, umeclidinium/vilanterol and tiotropium/olodaterol, it was shown that the patients preferred Ellipta^®^ over Respimat^®^ [44].

Nebulizers are not used on a daily basis for being more expensive, bulky and for taking more time for the dose inhalation; they are more often used during an exacerbation when patients may have trouble generating an adequate inspiratory flow.

Therefore, there are a great number of device characteristics that interfere with adherence and the HCP should understand the patients’ needs, the barriers that make adherence to treatment difficult and actively discuss better ways to improve the patient’s adherence. Inhaler device switching can lead to new or multiple device use and patients can face new challenges and barriers to this new treatment [11]. However, with new medications and devices being launched and multiple medication in one device, it will be necessary to evaluate the inhaler device switching according to patients’ characteristics (symptoms, disease severity and inhaler technique) to improve the adherence and individualize patients’ treatment. Multiple devices and mixing devices should lead patients to be less adherent and have higher treatment discontinuation [11].

## 6. Interventions to Improve Adherence and Inhaler Technique

Regardless of the non-adherence type, the important steps are to identify it, discuss this problem, reinforce the importance of the adherence and implement a better treatment adopted to the patient’s lifestyle. It is important to have an open and non-discriminative conversation and discussion to reach these objectives and to improve the COPD control. To improve the erratic non-adherence several techniques and technologies could be used: reduce the medication frequency (once daily dosing), simplification of the treatment (choose one inhaler device or prescribe as many medications as possible for the same time), link the medication to daily life activities (keep the inhaler on the nightstand or next to the toothbrush—check the device’s compatibility to bathroom humidity), memory aids (medication diary and pill organizers), and use of digital health technologies (smartphones apps, websites). It has been shown that patients frequently forget the instructions received during a clinic visit [5], and this may result from lack of communication between HCP and patient and contribute to the non-adherence to COPD treatments. In asthmatic patients it is common to mistake between as-needed medication (relief medication) and daily medication (maintenance medication) [45].

One interesting change in medication was the change of MDI to HFA after the Montreal Agreement made its use easier by patients as it allows longer puff duration, reducing the need for fine coordination between actuation and inhalation. Another interesting step that has changed after the HFA technique is that is not mandatory any longer to shake the canister before use. Nowadays, shaking is dependent on the type of the drug formulation, a solution or suspension [1]. An error usually described in the literature is the inadequate distance between the inhaler and the patient’s lips what can result in the medication impacting the oropharynx. This was a problem to worry about during the use of chlorofluorocarbon (CFC) gas due to its high flow. The way to circumvent this problem was either using a spacer or placing the inhaler at least 5 cm from a wide-open mouth. However, since the introduction of the HFA gas inhaler, its low flow means this rule is no longer mandatory. Patient education, simplicity of the device operation and the best adaptation of the patient to the inhaler should guide the physician in prescribing the device [46]. 

Besides the factors that lead to poor adherence, we should work to improve the adherence and inhaler technique. Poor inhaler technique is associated with poor control of respiratory diseases and consequent poor treatment adherence. The problems with the device use by the patients have been noted following the launch of the MDI. A recent systematic review about the errors on device technique demonstrated that these errors are frequent in both MDI and DPI use [47]. The overall rate of correct technique was 31%. The most frequent errors in MDI use were coordination (45%), inspiratory flow and depth of inspiration (44%) and no post inhalation breath hold (46%). The most frequent DPI errors were incorrect preparation (29%), no full expiration before inhalation (46%) and no post inhalation breath-hold (37%) [47].

Education of patients and their caregivers by HCPs plays an important role in inhaler use in order to minimize errors and optimize treatment [48]. A systematic review that evaluated the effectiveness of educational inhaler technique interventions in asthma and COPD showed that 89% of the studies included a physical or video demonstration of inhaler use with mean duration session of 30 min. Over 90% of the studies reported a significant improvement in the inhaler technique and the majority indicated favorable results for clinical outcomes (symptoms, health care utilization, quality of life and lung function) [49]. Ideally, all patients should be asked to use their inhalers during every health professional visit. So, the possible errors could be corrected, besides allowing the identification of the profile of those patients who require further clarification regarding inhaler use. A single-blind randomized trial that evaluated the impact of community pharmacist interventions on pharmacotherapeutic monitoring of COPD patients showed that the inhalation technique and adherence were significantly higher in the intervention group compared to the control group. In addition, the patients had a reduction in hospitalization rate, showing that a multi-professional approach can improve the outcomes [50]. The assessment of inhaler technique knowledge among HCPs reveals limited expertise among nurses, doctors, and respiratory therapists. In a systematic review it was demonstrated that historically, HCPs had inadequate knowledge about the correct use of inhalers, but there have been improvements in the last years. Between 1975–1995, the inadequate inhaler technique was 20.5% and between 1996–2014 there was a reduction to 10.8%. This poor knowledge could lead to less effective communication to patients and impact on the patient related outcomes [51]. This study showed that inhaler technique education must be for all patients and for the HCPs that do not have frequent access to respiratory training. 

## 7. Conclusions

COPD documents and guidelines underline the importance of correct inhaler technique training and frequent inhaler technique checks. This is because non-adherence to COPD medication worsens clinical and economic outcomes, making non-adherent patients a priority for cost-effective interventions. To improve adherence, the therapeutic decisions should be discussed with the patient and should take into consideration their lifestyle factors, demographic characteristics (age, co-morbidities, physical limitations, psychological and cognitive status), and pharmacological factors (polypharmacy regimens) to choose the best inhaler device for that patient. Therefore, the treatment choice and device can be tailored to individual patient needs and preferences. In addition, personalized interventions to correct the factors that lead to non-adherence and/or incorrect inhaler technique should be provided to improve health benefits among COPD patients.

## Figures and Tables

**Table 1:** Advantages and disadvantages of inhaler devices.

Device Type	Advantages	Disadvantages
Pressurized metered dosing inhaler (pMDI)	Multi-dose Compact Less expensive Fast to use	Perfect hand-mouth coordination Higher oropharyngeal deposition No dose counter
Dry powder inhaler (DPI)	Less coordination needed Compact Fast to use Less drug deposition	After drug preparation it is needed to use Drug preparation is different among each DPI device Higher inspiratory flow
Soft mist inhaler (SMI)	Compact Low drug flow	Difficult to prepare the device for first use Daily dose preparation difficulty Small dose counter numbers Requires more coordination for inhalation
Nebulizers	Treatment with many drugs and individualize the dose of each drug No coordination needed	Bulky Need for power source Long time to treatment Higher dose needed due to wasting drug and chance to more side effects Maintenance and cleaning Variation between the models Expensive
